# The association of smoking and demographic characteristics on body mass index and obesity among adults in the U.S., 1999–2012

**DOI:** 10.1186/s40608-014-0018-0

**Published:** 2014-08-30

**Authors:** Nantaporn Plurphanswat, Brad Rodu

**Affiliations:** James Graham Brown Cancer Center, University of Louisville, 505 South Hancock Street, Louisville, KY 40202 USA; Department of Medicine, School of Medicine, University of Louisville, 505 South Hancock Street, Louisville, KY 40202 USA

**Keywords:** Smoking, BMI, Obesity, NHANES

## Abstract

**Background:**

The National Health and Nutrition Examination Surveys (NHANES) are an exceptional data source for studies of smoking and body weight because they are the only federal survey series collecting relevant information through detailed interviews and medical examinations. The associations of smoking status and demographic factors with body weight have not been evaluated fully in recent NHANES.

**Methods:**

Using NHANES datasets from 1999 to 2012, this study uses ordinary-least squares and ordered probit models to investigate the association of smoking and selected demographic variables with body mass index (BMI) and the probability of being in BMI categories among adults aged 25–64 years, and it uses quantile regression to examine whether these factors affect individuals differently depending on where they are located across the BMI distribution.

**Results:**

The sample consisted of 11,123 men and 10,949 women. Current smokers had significantly lower BMI than never smokers (1.97 unit for men and 1.46 unit for women), and there was modest variation across the BMI distribution. Among former smokers, only women had a slightly higher BMI compared to never smokers (0.46 unit). Both men and women current smokers were more likely to be underweight and normal weight compared to never smokers and were less likely to be obese. Among men a one-year age increase elevated BMI by 0.2 unit throughout the BMI distribution, while for women an extra year of age increased BMI at the upper tail of the distribution more than at the lower tail. Education beyond high school was associated with a significant decrease in BMI among women, but much less so among men. Married men had higher BMI, but married women had significantly lower BMI, and this difference became larger at the upper tail.

**Conclusions:**

Compared to never smokers, men and women current smokers had lower BMI and lower probability of obesity, while only women former smokers had elevated BMIs and increased probability of obesity. In addition, we found that age, education and marital status were associated with different effects on BMI in men and women.

## Background

While the prevalence of cigarette smoking has substantially declined since the 1970s [1], the proportion of the population who are obese (defined as body mass index, BMI, of 30 or more) has increased [2]. Tobacco contains nicotine, the consumption of which is associated with reduced body weight via appetite reduction, reduced caloric intake and increased metabolic rate [3-5].

Previous studies have found mixed evidence of the association between smoking and obesity [6-11]. But there is more consistent evidence from population studies that smoking cessation leads to substantial weight gain [12-14].

The main goal of the present study is to examine the associations between smoking and demographic factors and BMI using the most recent NHANES datasets from 1999 to 2012. This study also extends the existing literature by using quantile regression to examine whether smoking status and demographic factors affect individuals differently depending on where they are located across the BMI distribution.

## Methods

### Data and variables

We used public-access data files from the 1999–2012 National Health and Nutrition Examination Survey (NHANES), conducted by the National Center for Health Statistics (NCHS). The first NHANES was administered in 1971. Since 1999 the survey has been a continuous program, examining a nationally representative sample of about 5,000 persons each year.

The NHANES interview includes demographic, socioeconomic, dietary, and health-related questions. The examination component consists of medical, dental, and physiological measurements, as well as laboratory tests administered by highly trained medical personnel.

Body weight and height were obtained by trained health technicians at medical examination sites. BMI was calculated as body weight in kilograms divided by the square of height in meters (kg/m^2^). Following the National Institutes of Health in Clinical Guidelines [15], we classified subjects as underweight (BMI < 18.5), normal weight (18.5 ≤ BMI < 25), overweight (25 ≤ BMI < 30) or obese (BMI ≥ 30).

In NHANES questionnaires, each respondent was asked about smoking behavior. We defined subjects as never smokers (those who never smoked 100 cigarettes in their lifetime), current smokers (those who smoked at least 100 cigarettes in their lifetime and also smoked now), or former smokers (those who smoked at least 100 cigarettes in their lifetime but did not smoke now). Current smokers were asked to provide information about cigarette consumption, which we categorized according to cigarettes smoked per day (1–14, 15–24 and 25+). Former smokers were asked about the length of time since smoking cessation, which we categorized according to number of years since quitting (<1, 1–4, 5–9 and 10+).

We included a set of demographic variables associated with BMI: age, height, race and ethnicity (white, black, Hispanic, and other, which included American Indian, Alaska Native, Pacific Islander, and Asian), foreign or U.S. born, highest educational attainment (less than high school, high school, more than high school), marital status (married and unmarried) and number of children (women only). In addition, we included a quadratic of age (age squared) to capture a non-linear relationship between that variable and BMI. We also controlled for survey cycle (1999–2000, 2001–02, 2003–04, 2005–06, 2007–08, 2009–10, and 2011–12), and we constructed sample weights across survey cycles using a formula from NCHS guidelines [16].

Notably, participants who were in a single person household during part of the 1999–2000 cycle were not asked about their marital status [15], and it was subsequently imputed by NHANES for most of them. However, it remained undetermined for about 300 subjects, in which we created an indicator with the value of 1 for respondents with missing marital status and 0 otherwise. We did the same for women who had no information on their pregnancy history. In this manner subjects with missing information were not excluded from the analyses and were subject to full evaluation.

We restricted our sample to adults aged 25 to 64 years. We excluded respondents aged 18–24 years because of inconsistencies in the 100-cigarette lifetime question between surveys. We also excluded pregnant women and respondents aged 65+ years because both smoking and weight status may be influenced by chronic illnesses at older ages.

### Empirical models

In this study, we examined the associations between smoking status (i.e. never, current and former smokers) and both a continuous measure of BMI and conventional BMI categories. We employed quantile regression and an ordered probit model to study the association of smoking status and weight across the BMI distribution and across BMI categories.

First, we examined the association between smoking status and a continuous measure of BMI using the Ordinary-Least Squares (OLS) method using the following model:

1 $$ BMI={\beta}_0+{\beta}_1S+{\beta}_2X+{\beta}_3Z+\varepsilon $$

where S represents smoking status categories (never, current and former smokers); X is a vector of individual characteristics; Z is a vector of survey cycle; and ε is an error term.

The main coefficient of interest is *B*_*1*_, which captures the relationship between smoking status on BMI. Positive or negative coefficients on current or former smokers indicate that, compared to never smokers, these groups have lower or higher BMI. Next, the vector X includes demographic variables such as age, race and ethnicity, US or foreign born, education, and marital status. A set of coefficients *β*_2_ present the associations of these variables and BMI. Finally, the vector Z refers to survey cycles, which allows us to account for unobservable confounding variables that may vary across survey cycles.

While the OLS regression estimates the association of smoking with average BMI, this relationship may differ across the BMI distribution [17]. For example, compared to never smokers, being a current or former smoker may reduce or increase BMI differently among those who are in the upper tail of the distribution compared with those in the lower tail. Therefore, we also employed the quantile regression (QR) analysis, which allows us to examine the entire conditional distribution of BMI and determine if an association between smoking and demographic variables differed across the BMI distribution. We estimated the following model:

2 $$ BMI={\beta}_0^p+{\beta}_1^pS+{\beta}_2^pX+{\beta}_3^pZ+{\varepsilon}^{\left(\mathrm{p}\right)} $$

where p refers to the proportion of the population having BMI below the quantile at *p*. In this study, *p* represents five quantiles: 10th, 25th, 50th, 75th and 90th. ${\beta}_1^p$ represents an association between smoking and BMI for the *p*^*th*^ conditional quantile.

The relationship between smoking and BMI may be non-linear, so we estimated an association between smoking status and BMI categories, CBMI (underweight = 1, normal weight = 2, overweight = 3, and obese = 4) by using the ordered probit (OP) model. In a nutshell, we estimated the following model:

3 $$ CBMI={\beta}_0+{\beta}_1S+{\beta}_2X+{\beta}_3Z+\varepsilon $$

However, the coefficients of OP estimates cannot be interpreted directly as the coefficients of linear models. We calculated the marginal effects, which measures the changes in probability of being underweight, normal, overweight or obese associated with a change from a never smoker to a current or former smoker with all other independent variables held at the values of their means.

## Results

### Descriptive statistics

The total sample consisted of 11,123 males and 10,949 females with complete information on smoking and BMI (Table 1). The average BMI was 28.6 for men and 28.8 for women. About 1-2% of men and women were underweight. Compared to women, a lower proportion of men had normal weight (26% vs. 34%) but more were overweight (40% vs. 28%). More men were also current (28% vs. 23%) and former smokers (25% vs. 20%). Women were about a year older on average than men in our sample (44.1 vs. 43.4). The majority of respondents were white, and about 20% were foreign born. Over 80% at least a high school diploma and most were married. Over 70% of women in our sample had a history of childbirth, with the majority having 1–2 children.

**Table 1:** **Summary of weighted descriptive statistics of analysis variables by sex, NHANES 1999-2012**

	**Men**		**Women**	
	**Mean**	**95% CI**	**Mean**	**95% CI**
Weight in lbs	196.48	[195.19, 197.78]	168.06	[166.76, 169.37]
Height in cm	176.54	[176.32, 176.76]	162.82	[162.62, 163.01]
Body Mass Index (BMI)	28.59	[28.42, 28.77]	28.80	[28.59, 29.02]
Weight categories (%)				
Underweight	1%	[1,1]	2%	[2, 2]
Normal weight	26	[25, 27]	34	[32, 35]
Overweight	40	[39, 42]	28	[27, 29]
Obese	33	[32, 34]	36	[35, 38]
Smoking status (%)				
Never smokers	47%	[45, 48]	57%	[56, 59]
Current smokers	28	[27, 30]	23	[22, 24]
Number of cigarettes/day (%)^1^				
1-14 cigarettes	46%	[44, 49]	52%	[49, 55]
15-24 cigarettes	34	[32, 36]	35	[33, 37]
25 + cigarettes	19	[17, 22]	13	[11, 15]
Former smokers	25	[24, 27]	20	[19, 21]
Years since quit smoking (%)^2^				
Less than 1 year	9%	[8, 10]	9%	[7, 11]
1-4 years	16	[16, 18]	17	[15, 19]
5-9 years	16	[14, 18]	15	[14, 17]
10 + years	59	[56, 61]	58	[55, 62]
Age	43.35	[43.02, 43.67]	44.12	[43.82, 44.42]
Race/Ethnicity (%)				
White	69%	[67, 72]	69%	[65, 71]
Black	11	[9, 12]	12	[11, 14]
Hispanic	14	[12, 16]	13	[11, 15]
Other	6	[5, 7]	6	[5, 7]
Foreign born (%)	19%	[17, 21]	17%	[15, 18]
Education (%)				
< High school	18%	[17, 19]	17%	[16, 18]
High school	25	[23, 26]	23	[22, 24]
> High school	57	[55, 59]	60	[59, 62]
Married (%)	70%	[69, 72]	66	[65, 68]
Missing marital status	2	[1, 3]	1	[1, 2]
Number of kids (%)				
No children	n/a	n/a	16%	[15, 18]
1-2 children	n/a	n/a	46	[44, 46]
3-4 children	n/a	n/a	24	[23, 25]
5 or more children	n/a	n/a	4	[4, 5]
Missing	n/a	n/a	10	[9, 11]
Observations	11123		10949	

^1^the proportions for number of cigarettes per day were derived from current smokers (3,359 men and 2,392 women).

^2^ the proportions for years since quit smoking were derived from former smokers (2,791 men and 1,901 women).

n/a – not applicable.

### Associations between BMI, smoking and demographic characteristics

The OLS results are presented in Table 2. Among men, current smokers had a significantly lower BMI than never smokers by 1.97 unit (about 6.9% lower than the mean), while former smokers were not different. Women smokers also had a significantly lower BMI than never smokers (1.49 unit), and the BMI among former smokers was elevated by 0.46 unit.

**Table 2:** **Associations between BMI and smoking status and demographic characteristics**

	**Men**		**Women**	
	**Coefficient**	**Std. error**	**Coefficient**	**Std. error**
Smoking status (ref. never smokers)				
Current smokers	−1.97^**^	(0.13)	−1.49^**^	(0.19)
Former smokers	0.01	(0.14)	0.46^*^	(0.19)
Height in centimeters	0.02^**^	(0.01)	−0.05^**^	(0.01)
Age	0.22^**^	(0.04)	0.32^**^	(0.05)
Age^2^	−0.00^**^	(0.00)	−0.00^**^	(0.00)
Race/Ethnicity (ref. white)				
Black	0.47^*^	(0.20)	2.76^**^	(0.25)
Hispanic	1.55^**^	(0.21)	1.28^**^	(0.26)
Other	−0.89^**^	(0.25)	−1.75^**^	(0.32)
Foreign born	−2.40^**^	(0.15)	−2.67^**^	(0.20)
Education (ref. < high school)				
High school	0.27	(0.17)	−0.33	(0.22)
> High school	−0.35^*^	(0.15)	−1.14^**^	(0.19)
Marital Status (ref. unmarried)				
Married	0.72^**^	(0.13)	−0.74^**^	(0.15)
Missing marital status	0.07	(0.42)	−0.27	(0.62)
Number of children (ref. 0 children)				
1-2 children	n/a	n/a	−0.18	(0.25)
3-4 children	n/a	n/a	0.14	(0.27)
5 or more children	n/a	n/a	0.42	(0.36)
Missing	n/a	n/a	−0.72^*^	(0.30)
Mean BMI	196.48		168.06	
Observations	11123		10949	

*p < 0.05, **p < 0.01.

All models include survey dummy variables and sample weights.

ref. – referent group.

n/a – not applicable.

Some demographic variables had a significant relationship with BMI. An increase in one year of age increased BMI by 0.22 units in men and 0.32 units in women. Blacks and Hispanics had higher BMI by 0.47 and 1.55 units in men and 2.76 and 1.28 units in women. BMI was lower among foreign born men (2.40 units) and women (2.67 units). Education beyond high school was associated with lower BMI among men (0.35 units) and women (1.14 units). While married men had higher BMI (0.72 units), married women had lower BMI of the same magnitude (0.75 units).

### Associations between BMI, smoking and demographic characteristics across the BMI distribution

Table 3 contains the QR estimates for current and former smokers, controlling for other characteristics, compared with never smokers. Among men, current smokers had significantly lower BMI than never smokers of about 1.6 to 1.75 units up to the 75th percentile; at the upper tail of 90th percentile they were 2.37 units lower. Former smokers did not have any statistically significant differences throughout the BMI distribution. Among women, current smokers had significantly lower BMI of about 1.02 to 1.65 units at all percentiles. Women former smokers had modest but significantly higher BMI throughout the distribution except at the 90th percentile, ranging from 0.38 to 0.75.

**Table 3:** **Selected associations between smoking status and BMI distributions**

	**Men**			**Women**		
	**BMI** ^**a**^	**Coefficients**		**BMI** ^**a**^	**Coefficients**	
	**Never smokers**	**Current smokers**	**Former smokers**	**Never smokers**	**Current smokers**	**Former smokers**
10th percentile	22.82	−1.75^**^	0.21	21.5	−1.13^**^	0.38^*^
		(0.16)	(0.17)		(0.18)	(0.19)
25th percentile	25.17	−1.70^**^	0.18	24.18	−1.02^**^	0.50^**^
		(0.13)	(0.13)		(0.17)	(0.18)
50th percentile	28.12	−1.60^**^	0.11	28.46	−1.18^**^	0.58^*^
		(0.13)	(0.14)		(0.21)	(0.22)
75th percentile	31.9	−1.74^**^	0.09	33.6	−1.65^**^	0.75^*^
		(0.20)	(0.21)		(0.46)	(0.30)
90th percentile	36.77	−2.37^**^	−0.21	39.6	−1.44^**^	0.90
		(0.32)	(0.34)		(0.28)	(0.49)

^a^BMI for each quantile.

Standard errors in parentheses; * p < 0.05, ** p < 0.01.

All models include age age^2^, race/ethnicity, foreign born, education attainment, marital status, number of children (female only), survey cycle, and sample weights.

**Figure 1:**
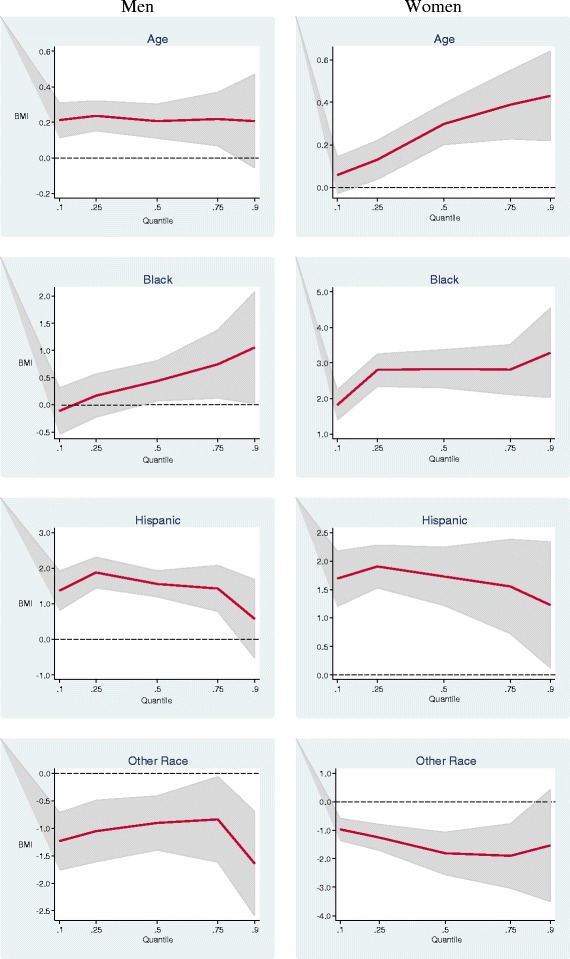
**Estimated multivariate coefficients for age and race/ethnicity on BMI using quantile regression.**

**Figure 2:**
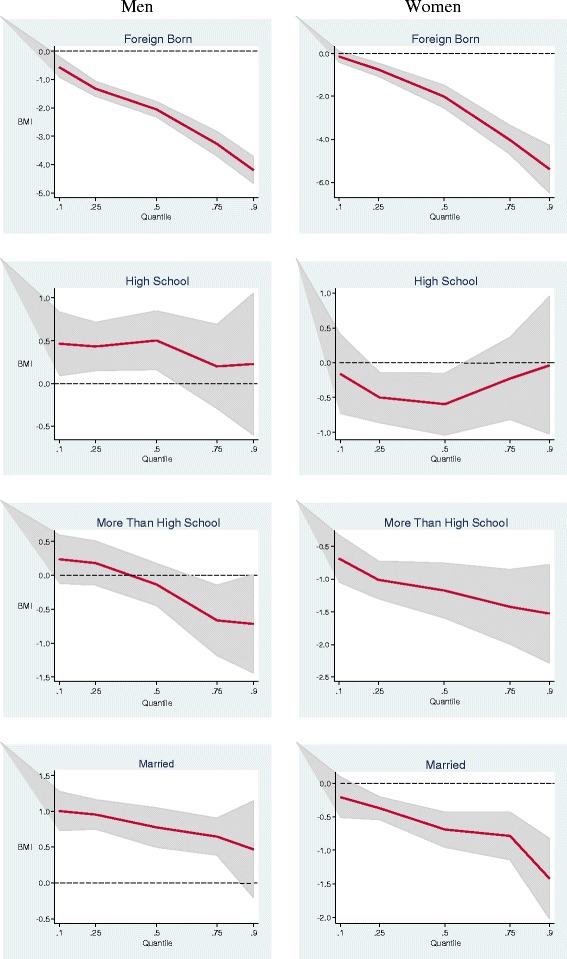
**Estimated multivariate coefficients for birthplace, education and marital status on BMI using quantile regression.**

Figures 1 and 2 present a summary of QR results for other selected variables among men and women. For each variable, the 5 QR estimates for specific percentiles from 0.10 to 0.90 are plotted as a solid curve, and the shaded gray area depicts the 95 percent confidence interval for the QR estimates. The dash lines represent the y-axis reference line at 0.

In Figure 1, as age increased by one year among men, the BMI increased by 0.2 unit throughout the BMI distribution, which was consistent with the OLS estimates. Among women, an extra year of age at the upper tail of the BMI distribution was associated with a larger increase. For example, at the 25th percentile a one-year increase in age increased BMI by 0.14 unit, but at the 75th percentile one year increased BMI by 0.43 unit. The disparity between white and black men was significantly larger in the upper half of the distribution. The disparity among black women was larger and significant throughout the BMI distribution. Hispanic men and women had higher BMIs than whites throughout the BMI distribution, whereas men and women of other races had BMIs consistently at least 1 unit lower.

Men and women who were born in foreign countries had significantly lower BMI than those who were born in the U.S., and this disparity sharply increased in the upper tail (Figure 2). Men who were high school graduates had higher BMI than those with less than high school throughout the distribution, but the association was significant only in the lower tail. Women who were high school graduates had lower BMI than those with less than high school throughout the distribution of BMI, but the relationship was significant only at the 25th and the 50th percentile. Among men, education beyond high school was associated with a significant decrease in BMI only in a narrow range near the 75th percentile, but in women it was associated with a significant decrease in BMI across the distribution. Married men had higher BMI than unmarried men, and this difference decreased at the upper tail. Married women had significantly lower BMI than unmarried women, and this difference became larger at the upper tail.

### Probability of BMI categories by smoking status

Table 4 shows the marginal effects of smoking status on weight categories by using an ordered probit regression model. Among men, a current smoker had a significantly higher probability of being underweight and normal weight by 0.9%, 11.6% respectively, and a lower probability of being obese by 12.7%, compared to a never smoker. Similar to the OLS and QR estimates, we found no difference between never smokers and former smokers in terms of being in particular BMI categories.

**Table 4:** **Probability of BMI categories by smoking status**

	**Men**		**Women**	
	**Current smoker**	**Former smoker**	**Current smoker**	**Former smoker**
Probability				
Underweight	0.0090^**^	−0.0003	0.0080^**^	−0.0024^*^
	(0.00)	(0.00)	(0.00)	(0.00)
Normal weight	0.1155^**^	−0.0043	0.0650^**^	−0.0225^*^
	(0.01)	(0.01)	(0.01)	(0.01)
Overweight	0.0029	−0.0005	0.0058^**^	−0.0038^*^
	(0.00)	(0.00)	(0.00)	(0.00)
Obesity	−0.1273^**^	0.0051	−0.0789^**^	0.0286^*^
	(0.01)	(0.01)	(0.01)	(0.01)

Standard errors in parentheses; * p < 0.05, ** p < 0.01.

All models include age age^2^, race/ethnicity, foreign born, education attainment, marital status, number of children (female only), survey cycle, and sample weights.

Among women, being a current smoker was associated with an increase in the probability of underweight (0.8%), normal weight (6.5%), and overweight (0.6%) and a decline in the probability of obesity (7.9%). Consistent with the OLS and QR estimates, a former smoker had lower probability of underweight (0.2%), normal weight (2.3%), and overweight (0.4%) and higher probability of obesity (2.9%).

We further explored the plausibility of the association between smoking and weight by examining cigarette consumption among current smokers and years since quitting among former smokers. We found no significant relationship between cigarette consumption and body weight among smokers. Among men former smokers, years since quitting was not associated with body weight. On the other hand, compared with women former smokers who quit more than 10+ years earlier, the probability of being obese increased by 11.2% in former smokers who quit 1–4 years earlier and increased by 6.5% in those who quit 5–9 years earlier.

## Discussion

Our results indicated differences in the relationship between smoking status and BMI according to sex. While current smoking among both men and women was associated with lower BMI throughout its distribution, the reduction among men who had very high BMI was greater than among those who had low and moderate values (2.37 units lower versus 1.7 unit respectively). In addition, being a smoker increased the probability of being normal weight by 12% and reduced the probability of being obese by 13%. The magnitudes for women were smaller than men, as the probability of being normal weight inclined by 7% while the probability of being obese decreased by 8%.

One plausible explanation for lower BMI and probability of being obese among smokers was that cigarettes may suppress appetite or increase metabolism [4]. Although previous European studies showed that cigarette consumption was positively associated with BMI [18,19] and obesity [19,20], we found no significant relationship.

Sex differences were also seen in former smokers. While weight gain after smoking cessation may be a disincentive to some smokers [21–23], we found no evidence of increased BMI among men former smokers, which is in contrast with a British longitudinal study [24]. On the other hand, women former smokers had modestly but significantly higher BMI than never smokers throughout the BMI distribution. The association of former smoking and obesity was weak. Compared to long-term (10+ years) quitters, women quitting 1 to 4 years earlier had a 11.2% increase in the probability of being obese. This result was comparable to a study of smoking cessation and weight in Denmark, in which women former smokers had gained more weight than men [11].

We found that the associations between demographic characteristics and BMI were different by sex. For example, while a one-year increase in age was associated with about 0.2 unit increase in BMI among men, an extra year of age among women increased BMI by 0.14 unit at the 25th percentile and 0.43 unit at the 75th percentile. Women with higher education had significantly lower BMI throughout the distribution than those with lower education; this relationship was not present among men. Interestingly, we found that marriage was associated with higher BMI among men but lower BMI among women. This finding is consistent with recent studies that used NHANES 1999–2002 [25,26].

The strengths of this study included accurately measured body weight and height and the accuracy of self-reported smoking status, which was established by a study finding that serum cotinine, a marker of exposure to nicotine, was consistent with self-reported smoking status in an earlier NHANES cohort (1988–1994) [27]. In addition, the QR model allowed us to examine an association between smoking and demographic variables across the BMI distribution instead of only at the conditional mean of BMI, which is a limitation of OLS methods.

A limitation of NHANES public-use datasets is that they do not allow us to identify respondents’ states of residence, so we were not able to control for state fixed-effects or merge state policies that might be the basis for unobservable characteristics simultaneously correlated with both body weight and smoking. For example, state policies that aim to improve health outcomes may reduce both smoking and obesity at the same time. Furthermore, our analysis does not reconcile all possible causal pathways between smoking and BMI. For example, high BMI may be associated with smoking behavior; it is plausible that obese persons smoke in order to lower their weight, which would also lead to lower prevalence of obesity compared with never smokers.

While physical activity was a significant predictor of BMI, we did not include it in our models because of the plausible reverse causality between body weight and exercise. For example, heavy individuals may not be able to participate in any exercise and, since they are not physically active, they are obese. Finally, we did not include energy intake in this study. Even though NHANES has information on caloric intake, we found irreconcilable discrepancies. For example, the underweight population reported high caloric consumption and the obese population reported low caloric consumption.

## Conclusion

This study provides evidence that smoking was associated with lower BMI and a lower probability of obesity among men and women in the U.S. during the period 1999 to 2012. The association of former smoking and BMI or obesity is modest and limited to women.
